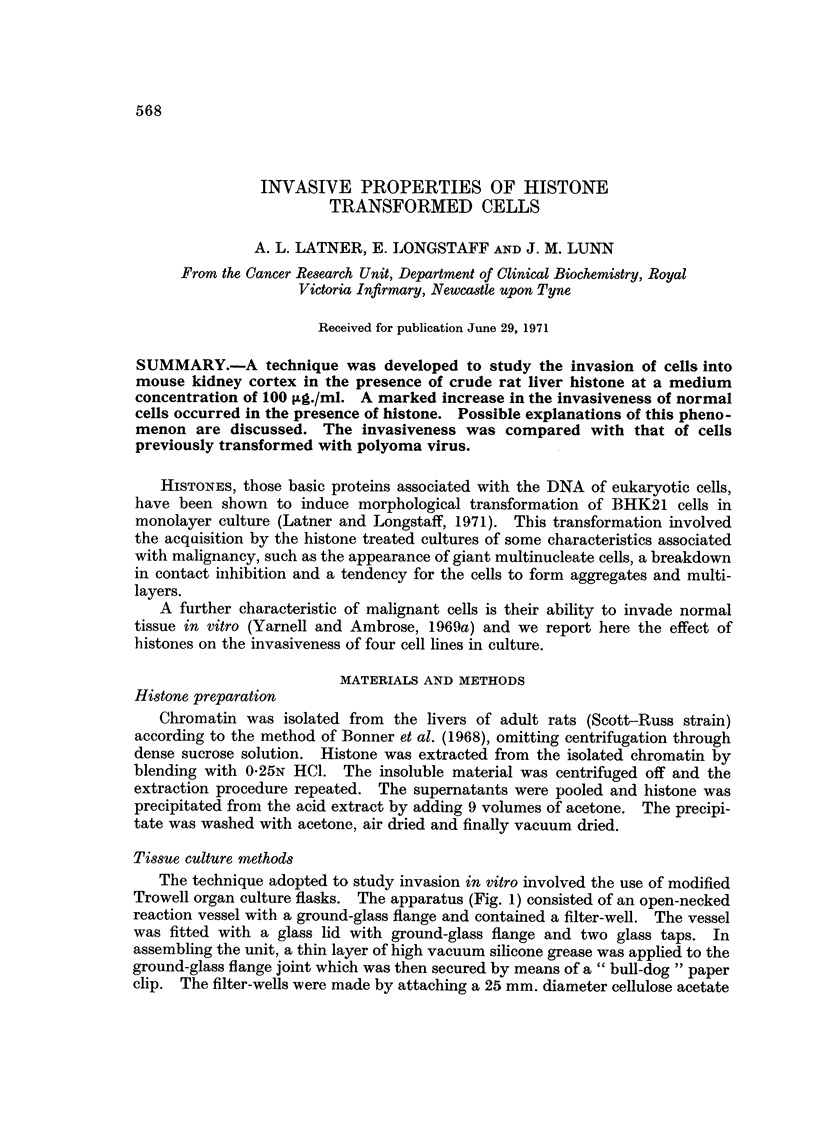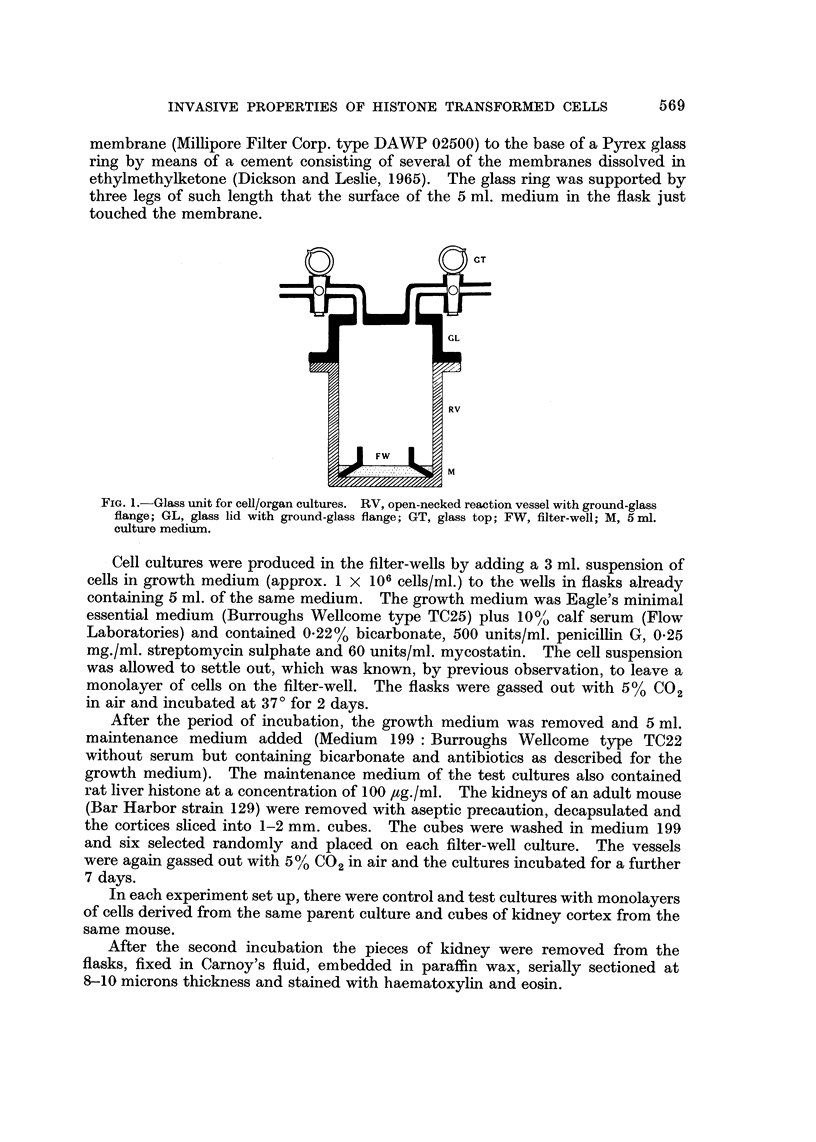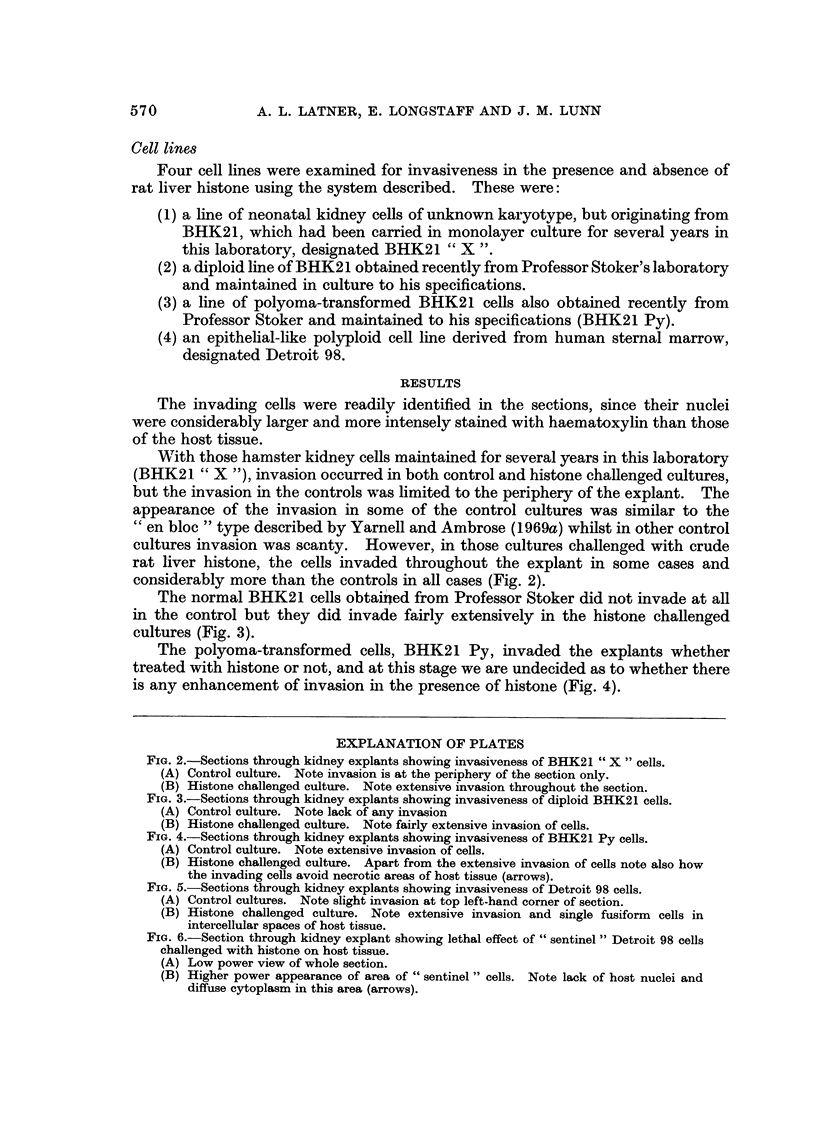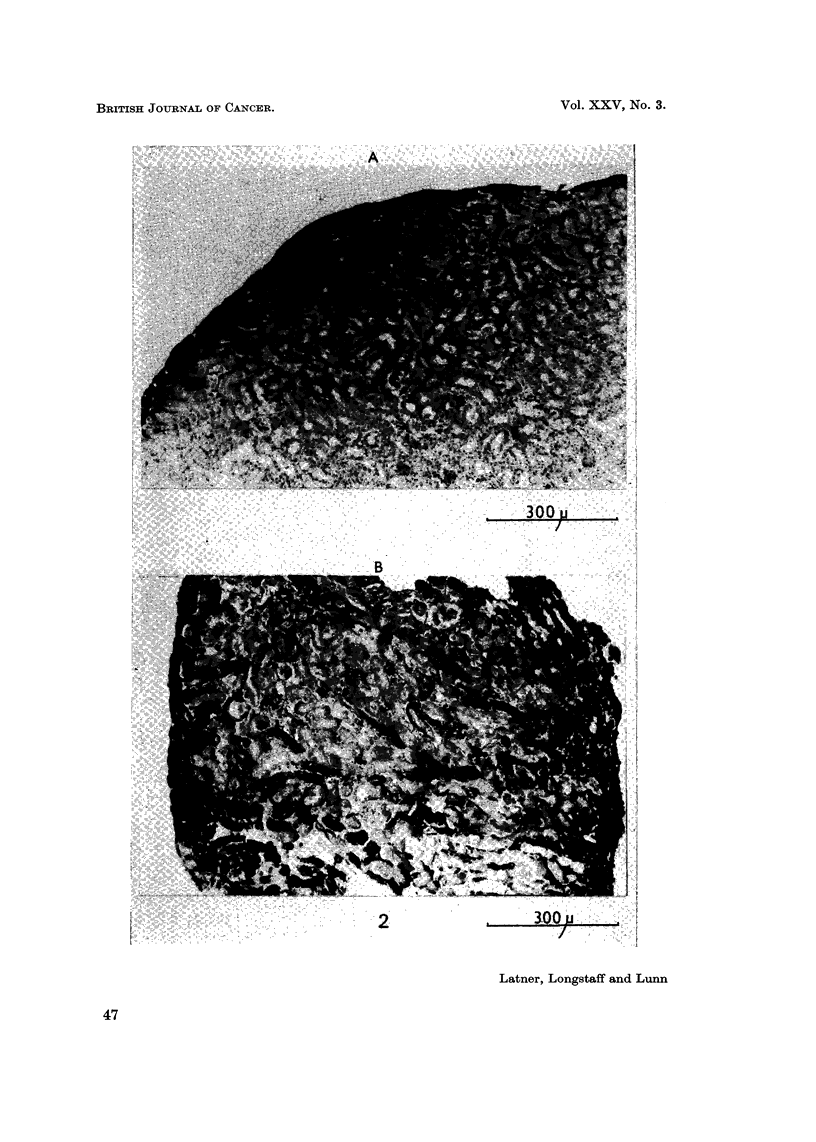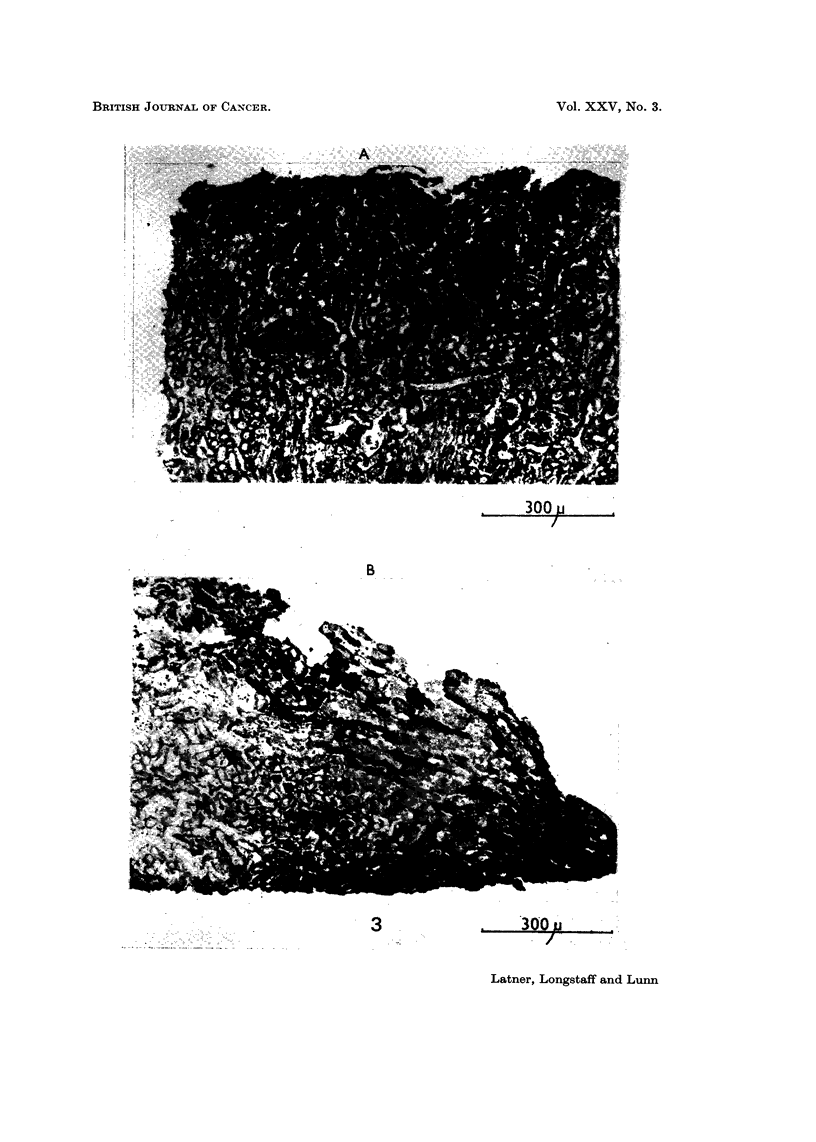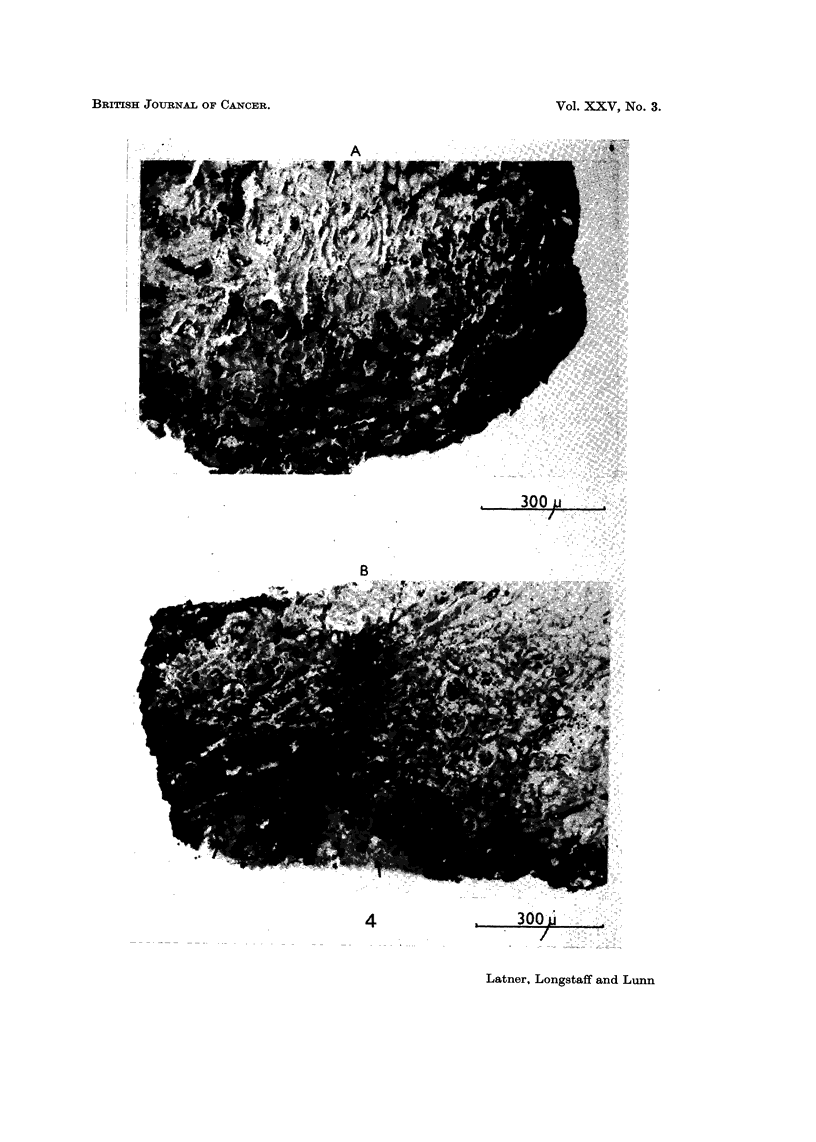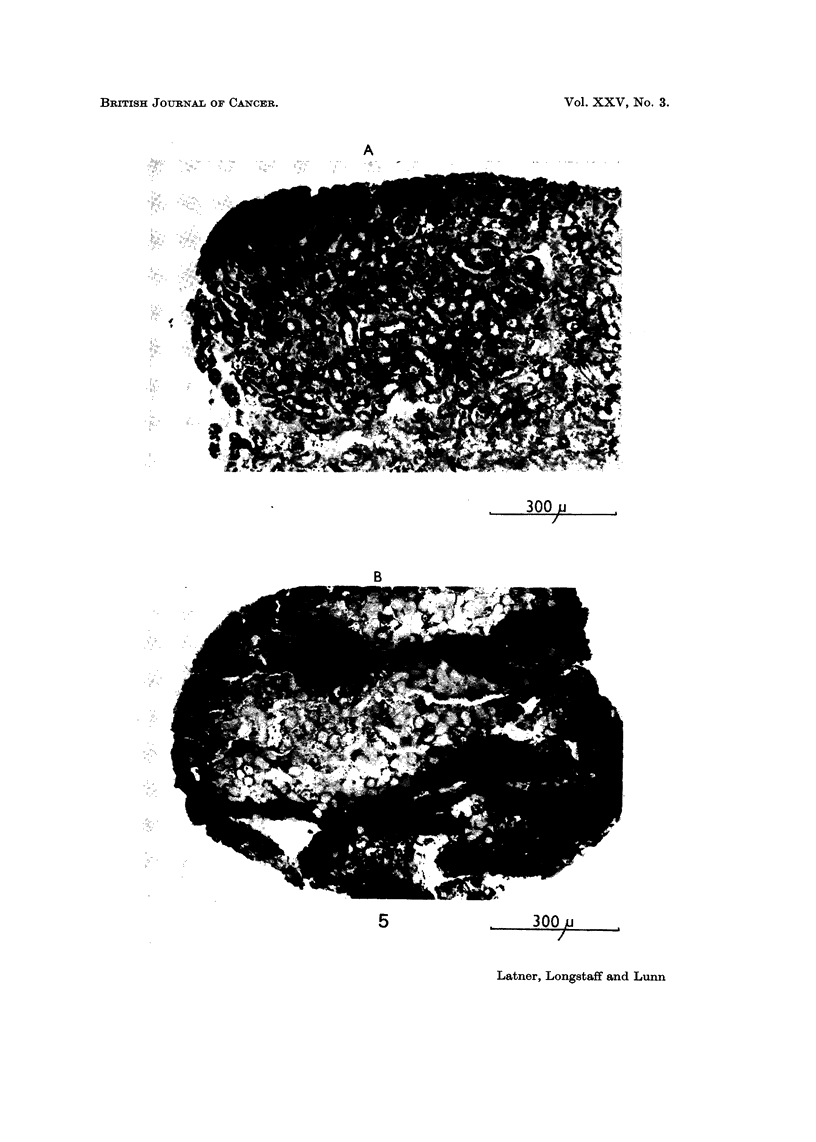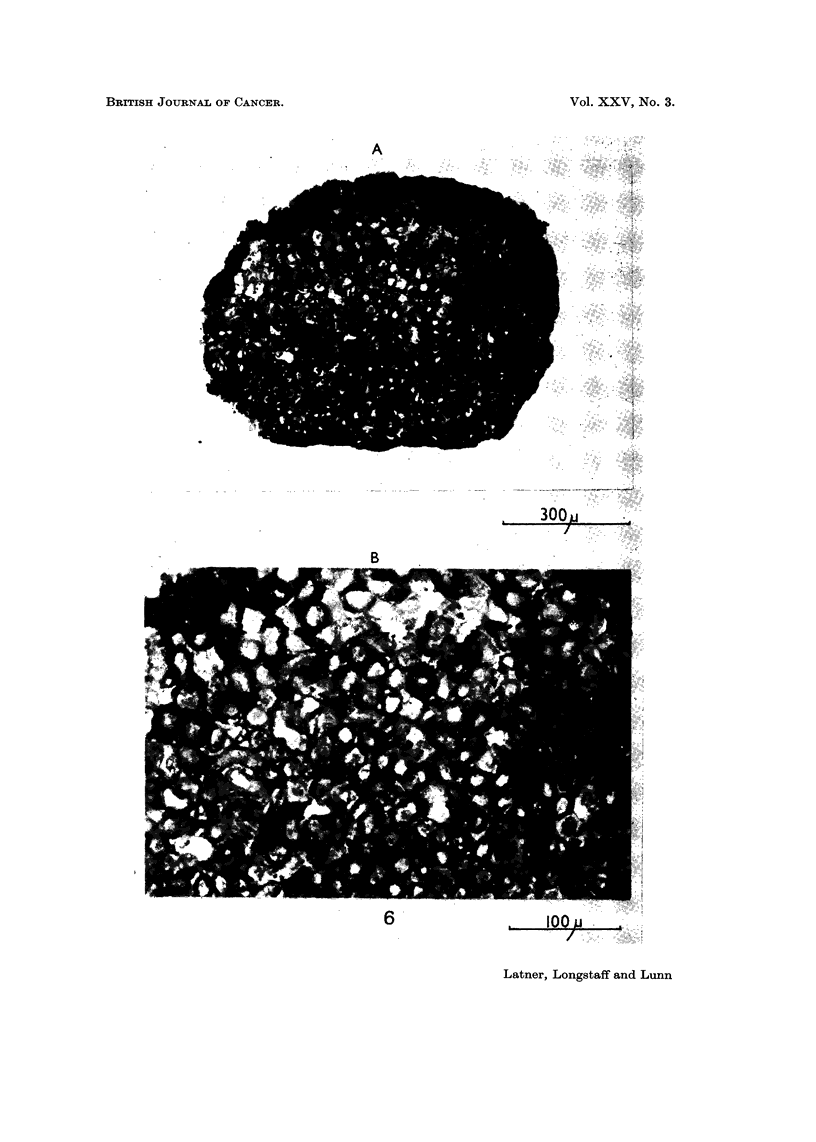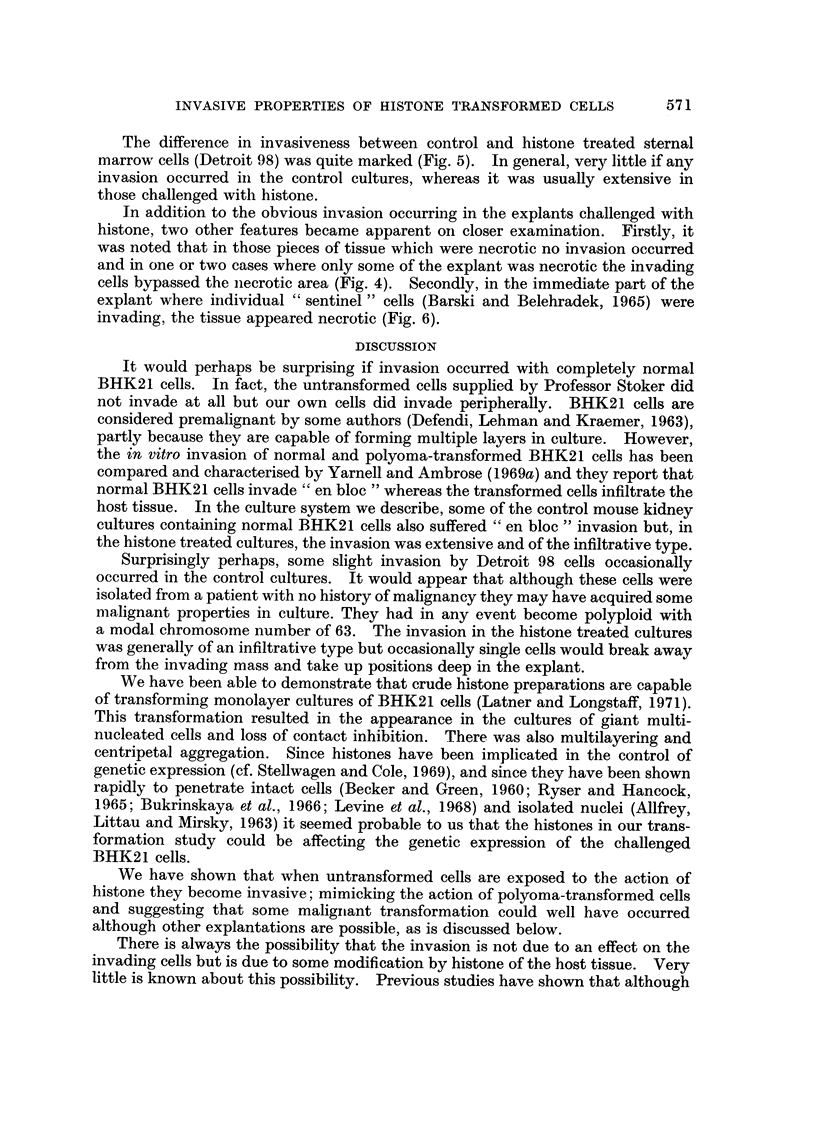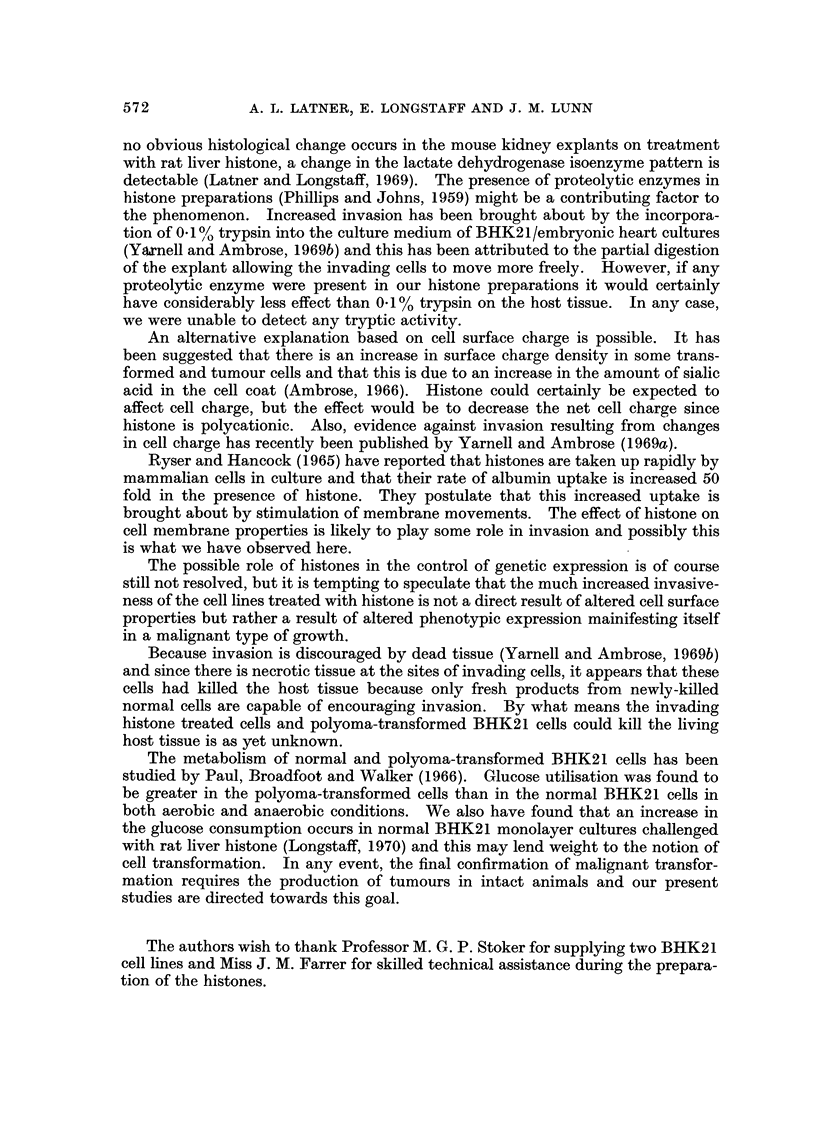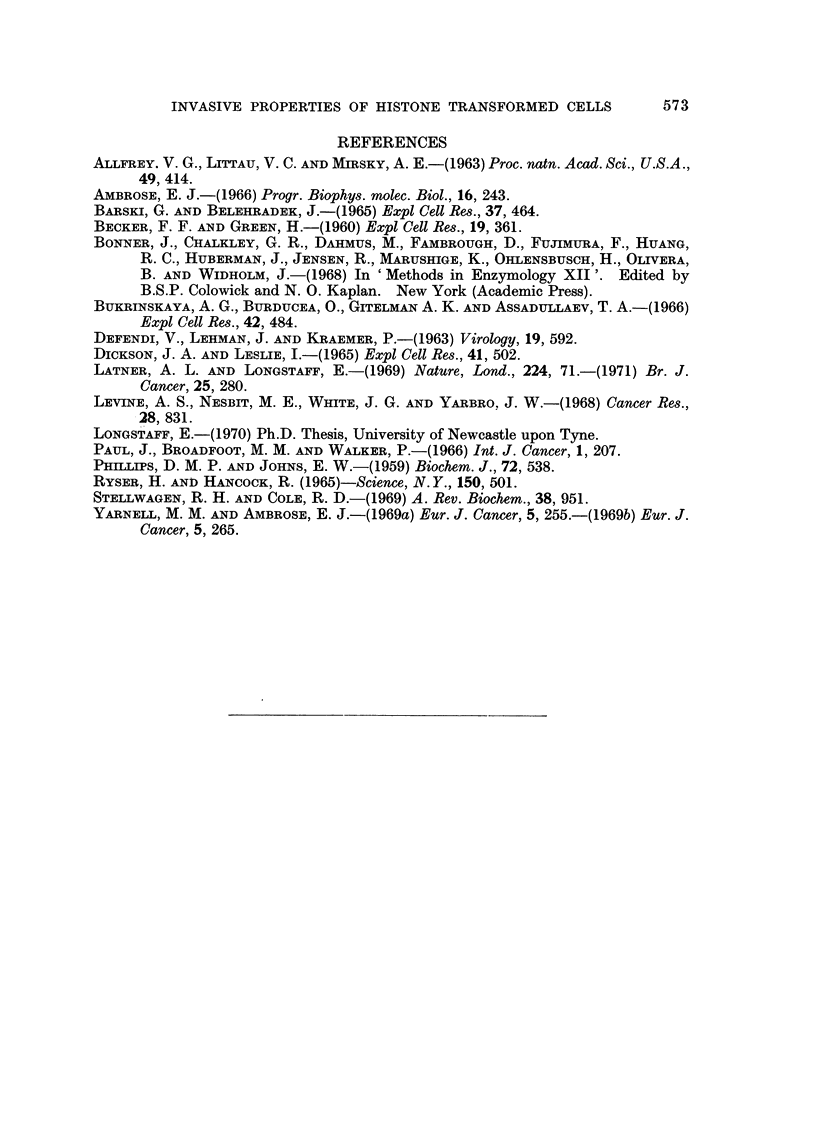# Invasive Properties of Histone Transformed Cells

**DOI:** 10.1038/bjc.1971.72

**Published:** 1971-09

**Authors:** A. L. Latner, E. Longstaff, J. M. Lunn

## Abstract

**Images:**


### INVASIVE PROPERTIES OF HISTONE TRANSFORMED CELLS

A. L. LATNER, E. LONGSTAFF AND J. M. LUNN

*From the Cancer Research Unit, Department of Clinical Biochemistry, Royal Victoria Infirmary, Newcastle upon Tyne*

Received for publication June 29, 1971

**SUMMARY.—A technique was developed to study the invasion of cells into mouse kidney cortex in the presence of crude rat liver histone at a medium concentration of 100 μg./ml. A marked increase in the invasiveness of normal cells occurred in the presence of histone. Possible explanations of this phenomenon are discussed. The invasiveness was compared with that of cells previously transformed with polyoma virus.**

HISTONES, those basic proteins associated with the DNA of eukaryotic cells, have been shown to induce morphological transformation of BHK21 cells in monolayer culture (Latner and Longstaff, 1971). This transformation involved the acquisition by the histone treated cultures of some characteristics associated with malignancy, such as the appearance of giant multinucleate cells, a breakdown in contact inhibition and a tendency for the cells to form aggregates and multilayers.

A further characteristic of malignant cells is their ability to invade normal tissue *in vitro* (Yarnell and Ambrose, 1969*a*) and we report here the effect of histones on the invasiveness of four cell lines in culture.

### MATERIALS AND METHODS

### *Histone preparation*

Chromatin was isolated from the livers of adult rats (Scott–Russ strain) according to the method of Bonner *et al.* (1968), omitting centrifugation through dense sucrose solution. Histone was extracted from the isolated chromatin by blending with 0·25N HCl. The insoluble material was centrifuged off and the extraction procedure repeated. The supernatants were pooled and histone was precipitated from the acid extract by adding 9 volumes of acetone. The precipitate was washed with acetone, air dried and finally vacuum dried.

### *Tissue culture methods*

The technique adopted to study invasion *in vitro* involved the use of modified Trowell organ culture flasks. The apparatus (Fig. 1) consisted of an open-necked reaction vessel with a ground-glass flange and contained a filter-well. The vessel was fitted with a glass lid with ground-glass flange and two glass taps. In assembling the unit, a thin layer of high vacuum silicone grease was applied to the ground-glass flange joint which was then secured by means of a " bull-dog " paper clip. The filter-wells were made by attaching a 25 mm. diameter cellulose acetate membrane (Millipore Filter Corp. type DAWP 02500) to the base of a Pyrex glass ring by means of a cement consisting of several of the membranes dissolved in ethylmethylketone (Dickson and Leslie, 1965). The glass ring was supported by three legs of such length that the surface of the 5 ml. medium in the flask just touched the membrane.

FIG. 1.—Glass unit for cell/organ cultures. RV, open-necked reaction vessel with ground-glass flange; GL, glass lid with ground-glass flange; GT, glass top; FW, filter-well; M, 5 ml. culture medium.

Cell cultures were produced in the filter-wells by adding a 3 ml. suspension of cells in growth medium (approx. $1 \times 10^6$ cells/ml.) to the wells in flasks already containing 5 ml. of the same medium. The growth medium was Eagle's minimal essential medium (Burroughs Wellcome type TC25) plus 10% calf serum (Flow Laboratories) and contained 0·22% bicarbonate, 500 units/ml. penicillin G, 0·25 mg./ml. streptomycin sulphate and 60 units/ml. mycostatin. The cell suspension was allowed to settle out, which was known, by previous observation, to leave a monolayer of cells on the filter-well. The flasks were gassed out with 5% $CO_2$ in air and incubated at 37° for 2 days.

After the period of incubation, the growth medium was removed and 5 ml. maintenance medium added (Medium 199 : Burroughs Wellcome type TC22 without serum but containing bicarbonate and antibiotics as described for the growth medium). The maintenance medium of the test cultures also contained rat liver histone at a concentration of 100 $\mu$g./ml. The kidneys of an adult mouse (Bar Harbor strain 129) were removed with aseptic precaution, decapsulated and the cortices sliced into 1–2 mm. cubes. The cubes were washed in medium 199 and six selected randomly and placed on each filter-well culture. The vessels were again gassed out with 5% $CO_2$ in air and the cultures incubated for a further 7 days.

In each experiment set up, there were control and test cultures with monolayers of cells derived from the same parent culture and cubes of kidney cortex from the same mouse.

After the second incubation the pieces of kidney were removed from the flasks, fixed in Carnoy's fluid, embedded in paraffin wax, serially sectioned at 8–10 microns thickness and stained with haematoxylin and eosin.

*Cell lines*

Four cell lines were examined for invasiveness in the presence and absence of rat liver histone using the system described. These were:

(1) a line of neonatal kidney cells of unknown karyotype, but originating from BHK21, which had been carried in monolayer culture for several years in this laboratory, designated BHK21 " X ".
(2) a diploid line of BHK21 obtained recently from Professor Stoker's laboratory and maintained in culture to his specifications.
(3) a line of polyoma-transformed BHK21 cells also obtained recently from Professor Stoker and maintained to his specifications (BHK21 Py).
(4) an epithelial-like polyploid cell line derived from human sternal marrow, designated Detroit 98.

### RESULTS

The invading cells were readily identified in the sections, since their nuclei were considerably larger and more intensely stained with haematoxylin than those of the host tissue.

With those hamster kidney cells maintained for several years in this laboratory (BHK21 " X "), invasion occurred in both control and histone challenged cultures, but the invasion in the controls was limited to the periphery of the explant. The appearance of the invasion in some of the control cultures was similar to the " en bloc " type described by Yarnell and Ambrose (1969*a*) whilst in other control cultures invasion was scanty. However, in those cultures challenged with crude rat liver histone, the cells invaded throughout the explant in some cases and considerably more than the controls in all cases (Fig. 2).

The normal BHK21 cells obtained from Professor Stoker did not invade at all in the control but they did invade fairly extensively in the histone challenged cultures (Fig. 3).

The polyoma-transformed cells, BHK21 Py, invaded the explants whether treated with histone or not, and at this stage we are undecided as to whether there is any enhancement of invasion in the presence of histone (Fig. 4).

---

### EXPLANATION OF PLATES

FIG. 2.—Sections through kidney explants showing invasiveness of BHK21 " X " cells.
(A) Control culture. Note invasion is at the periphery of the section only.
(B) Histone challenged culture. Note extensive invasion throughout the section.

FIG. 3.—Sections through kidney explants showing invasiveness of diploid BHK21 cells.
(A) Control culture. Note lack of any invasion
(B) Histone challenged culture. Note fairly extensive invasion of cells.

FIG. 4.—Sections through kidney explants showing invasiveness of BHK21 Py cells.
(A) Control culture. Note extensive invasion of cells.
(B) Histone challenged culture. Apart from the extensive invasion of cells note also how the invading cells avoid necrotic areas of host tissue (arrows).

FIG. 5.—Sections through kidney explants showing invasiveness of Detroit 98 cells.
(A) Control cultures. Note slight invasion at top left-hand corner of section.
(B) Histone challenged culture. Note extensive invasion and single fusiform cells in intercellular spaces of host tissue.

FIG. 6.—Section through kidney explant showing lethal effect of " sentinel " Detroit 98 cells challenged with histone on host tissue.
(A) Low power view of whole section.
(B) Higher power appearance of area of " sentinel " cells. Note lack of host nuclei and diffuse cytoplasm in this area (arrows).

Latner, Longstaff and Lunn

Latner, Longstaff and Lunn

Latner, Longstaff and Lunn

Latner, Longstaff and Lunn

Latner, Longstaff and Lunn

The difference in invasiveness between control and histone treated sternal marrow cells (Detroit 98) was quite marked (Fig. 5). In general, very little if any invasion occurred in the control cultures, whereas it was usually extensive in those challenged with histone.

In addition to the obvious invasion occurring in the explants challenged with histone, two other features became apparent on closer examination. Firstly, it was noted that in those pieces of tissue which were necrotic no invasion occurred and in one or two cases where only some of the explant was necrotic the invading cells bypassed the necrotic area (Fig. 4). Secondly, in the immediate part of the explant where individual " sentinel " cells (Barski and Belehradek, 1965) were invading, the tissue appeared necrotic (Fig. 6).

### DISCUSSION

It would perhaps be surprising if invasion occurred with completely normal BHK21 cells. In fact, the untransformed cells supplied by Professor Stoker did not invade at all but our own cells did invade peripherally. BHK21 cells are considered premalignant by some authors (Defendi, Lehman and Kraemer, 1963), partly because they are capable of forming multiple layers in culture. However, the *in vitro* invasion of normal and polyoma-transformed BHK21 cells has been compared and characterised by Yarnell and Ambrose (1969*a*) and they report that normal BHK21 cells invade " en bloc " whereas the transformed cells infiltrate the host tissue. In the culture system we describe, some of the control mouse kidney cultures containing normal BHK21 cells also suffered " en bloc " invasion but, in the histone treated cultures, the invasion was extensive and of the infiltrative type.

Surprisingly perhaps, some slight invasion by Detroit 98 cells occasionally occurred in the control cultures. It would appear that although these cells were isolated from a patient with no history of malignancy they may have acquired some malignant properties in culture. They had in any event become polyploid with a modal chromosome number of 63. The invasion in the histone treated cultures was generally of an infiltrative type but occasionally single cells would break away from the invading mass and take up positions deep in the explant.

We have been able to demonstrate that crude histone preparations are capable of transforming monolayer cultures of BHK21 cells (Latner and Longstaff, 1971). This transformation resulted in the appearance in the cultures of giant multinucleated cells and loss of contact inhibition. There was also multilayering and centripetal aggregation. Since histones have been implicated in the control of genetic expression (cf. Stellwagen and Cole, 1969), and since they have been shown rapidly to penetrate intact cells (Becker and Green, 1960; Ryser and Hancock, 1965; Bukrinskaya *et al.*, 1966; Levine *et al.*, 1968) and isolated nuclei (Allfrey, Littau and Mirsky, 1963) it seemed probable to us that the histones in our transformation study could be affecting the genetic expression of the challenged BHK21 cells.

We have shown that when untransformed cells are exposed to the action of histone they become invasive; mimicking the action of polyoma-transformed cells and suggesting that some malignant transformation could well have occurred although other explantations are possible, as is discussed below.

There is always the possibility that the invasion is not due to an effect on the invading cells but is due to some modification by histone of the host tissue. Very little is known about this possibility. Previous studies have shown that although no obvious histological change occurs in the mouse kidney explants on treatment with rat liver histone, a change in the lactate dehydrogenase isoenzyme pattern is detectable (Latner and Longstaff, 1969). The presence of proteolytic enzymes in histone preparations (Phillips and Johns, 1959) might be a contributing factor to the phenomenon. Increased invasion has been brought about by the incorporation of 0·1% trypsin into the culture medium of BHK21/embryonic heart cultures (Yarnell and Ambrose, 1969*b*) and this has been attributed to the partial digestion of the explant allowing the invading cells to move more freely. However, if any proteolytic enzyme were present in our histone preparations it would certainly have considerably less effect than 0·1% trypsin on the host tissue. In any case, we were unable to detect any tryptic activity.

An alternative explanation based on cell surface charge is possible. It has been suggested that there is an increase in surface charge density in some transformed and tumour cells and that this is due to an increase in the amount of sialic acid in the cell coat (Ambrose, 1966). Histone could certainly be expected to affect cell charge, but the effect would be to decrease the net cell charge since histone is polycationic. Also, evidence against invasion resulting from changes in cell charge has recently been published by Yarnell and Ambrose (1969*a*).

Ryser and Hancock (1965) have reported that histones are taken up rapidly by mammalian cells in culture and that their rate of albumin uptake is increased 50 fold in the presence of histone. They postulate that this increased uptake is brought about by stimulation of membrane movements. The effect of histone on cell membrane properties is likely to play some role in invasion and possibly this is what we have observed here.

The possible role of histones in the control of genetic expression is of course still not resolved, but it is tempting to speculate that the much increased invasiveness of the cell lines treated with histone is not a direct result of altered cell surface properties but rather a result of altered phenotypic expression mainifesting itself in a malignant type of growth.

Because invasion is discouraged by dead tissue (Yarnell and Ambrose, 1969*b*) and since there is necrotic tissue at the sites of invading cells, it appears that these cells had killed the host tissue because only fresh products from newly-killed normal cells are capable of encouraging invasion. By what means the invading histone treated cells and polyoma-transformed BHK21 cells could kill the living host tissue is as yet unknown.

The metabolism of normal and polyoma-transformed BHK21 cells has been studied by Paul, Broadfoot and Walker (1966). Glucose utilisation was found to be greater in the polyoma-transformed cells than in the normal BHK21 cells in both aerobic and anaerobic conditions. We also have found that an increase in the glucose consumption occurs in normal BHK21 monolayer cultures challenged with rat liver histone (Longstaff, 1970) and this may lend weight to the notion of cell transformation. In any event, the final confirmation of malignant transformation requires the production of tumours in intact animals and our present studies are directed towards this goal.

The authors wish to thank Professor M. G. P. Stoker for supplying two BHK21 cell lines and Miss J. M. Farrer for skilled technical assistance during the preparation of the histones.

### REFERENCES

ALLFREY. V. G., LITTAU, V. C. AND MIRSKY, A. E.—(1963) *Proc. natn. Acad. Sci., U.S.A.*, **49**, 414.

AMBROSE, E. J.—(1966) *Progr. Biophys. molec. Biol.*, **16**, 243.

BARSKI, G. AND BELEHRADEK, J.—(1965) *Expl Cell Res.*, **37**, 464.

BECKER, F. F. AND GREEN, H.—(1960) *Expl Cell Res.*, **19**, 361.

BONNER, J., CHALKLEY, G. R., DAHMUS, M., FAMBROUGH, D., FUJIMURA, F., HUANG, R. C., HUBERMAN, J., JENSEN, R., MARUSHIGE, K., OHLENSBUSCH, H., OLIVERA, B. AND WIDHOLM, J.—(1968) In 'Methods in Enzymology XII'. Edited by B.S.P. Colowick and N. O. Kaplan. New York (Academic Press).

BUKRINSKAYA, A. G., BURDUCEA, O., GITELMAN A. K. AND ASSADULLAEV, T. A.—(1966) *Expl Cell Res.*, **42**, 484.

DEFENDI, V., LEHMAN, J. AND KRAEMER, P.—(1963) *Virology*, **19**, 592.

DICKSON, J. A. AND LESLIE, I.—(1965) *Expl Cell Res.*, **41**, 502.

LATNER, A. L. AND LONGSTAFF, E.—(1969) *Nature, Lond.*, **224**, 71.—(1971) *Br. J. Cancer*, **25**, 280.

LEVINE, A. S., NESBIT, M. E., WHITE, J. G. AND YARBRO, J. W.—(1968) *Cancer Res.*, **28**, 831.

LONGSTAFF, E.—(1970) Ph.D. Thesis, University of Newcastle upon Tyne.

PAUL, J., BROADFOOT, M. M. AND WALKER, P.—(1966) *Int. J. Cancer*, **1**, 207.

PHILLIPS, D. M. P. AND JOHNS, E. W.—(1959) *Biochem. J.*, **72**, 538.

RYSER, H. AND HANCOCK, R. (1965)—*Science, N.Y.*, **150**, 501.

STELLWAGEN, R. H. AND COLE, R. D.—(1969) *A. Rev. Biochem.*, **38**, 951.

YARNELL, M. M. AND AMBROSE, E. J.—(1969*a*) *Eur. J. Cancer*, **5**, 255.—(1969*b*) *Eur. J. Cancer*, **5**, 265.